# Assessing VOC emissions from different gas stations: impacts, variations, and modeling fluctuations of air pollutants

**DOI:** 10.1038/s41598-024-67542-4

**Published:** 2024-07-18

**Authors:** Elham Alsadat Heidari, Maryam Sarkhosh, Hosein Alidadi, Ali Asghar Najafpoor, Habibollah Esmaily, Elham Shamsara

**Affiliations:** 1 Master of Environmental Health Engineering, Health Center of Torbat-e Heydarieh, Torbat-e Heydarieh University of Medical Sciences, Torbat-e Heydarieh, Iran; 2 Department of Environmental Health Engineering, School of Health, Mashhad University of Medical Sciences, Mashhad, Iran; 3 Department of Biostatistics, Research Health Center, School of Health, Mashhad University of Medical Sciences, Mashhad, Iran; 4 Management and Social Determinants of Health Research Center, Mashhad University of Medical Sciences, Mashhad, 91778-99191 Iran

**Keywords:** Volatile organic compounds, CNG station, Gasoline station, M,p-xylene, Modeling, Environmental monitoring, Pollution remediation, Environmental impact

## Abstract

Gas stations distributed around densely populated areas are responsible for toxic pollutant emissions such as volatile organic compounds (VOCs). This study aims to measure VOCs emission from three different kinds of gas stations to determine the extent of pollution from the gas stations and the most frequent type of VOC compound emitted. The concentrations of ambient VOCs at three refueling stations with a different type of fuels in Mashhad were monitored. The result of this study showed that CNG fuel stations are less polluting than petrol stations. In all the studied sites, the highest concentrations were related to xylene isomers, irrespective of the fuel type. Total VOCs at the supply of both compressed natural gas (CNG) and gasoline stations was 482.36 ± 563.45 µg m^−3^. At a CNG station and a gasoline station, total VOC concentrations were 1363.4 ± 1975 µg m^−3^ and 410.29 ± 483.37 µg m^−3^, respectively. The differences in concentrations of toluene and m,p-xylene between the fuel stations can be related to the quality and type of fuel, vapor recovery technology, fuel reserves, dripless nozzles, traffic density in these stations, meteorological conditions and the location of sampling sites. The combination of a sine function and a quadratic function could model the fluctuation behavior of air pollutants like m,p-xylene. In all the sites, the highest concentrations were related to xylene isomers, irrespective of the type of fuel. The changing rate of m,p-xylene pollutant in each station was also modeled in this study.

## Introduction

Volatile organic compounds (VOCs) are one of the most important pollutants in the atmosphere^1^. These compounds account for more than half of hazardous air pollutants^2–4^. VOCs play a significant role in stratospheric ozone depletion and the formation of toxic secondary pollutants; i.e., tropospheric ozone and peroxyacetyl nitrate (PAN)^5^. VOCs are classified as toxic pollutants with detrimental effects on human health, such as causing nose and throat irritation, exacerbating asthma, and possessing mutagenic and carcinogenic properties^4–6^. The effects of exposure to xylene can be divided into two types: acute effects and chronic effects. Acute effects include irritation of eyes, nose, and throat and damage to the lungs and nervous system. Chronic effects such as irritability, fatigue, headache, anemia, impaired short-term memory, kidney, and liver damage result from longe-term exposure^7–9^. Also researchers have found relationships between exposure to xylene and fatigue symptoms among students^10^. Volatile Organic Compounds (VOCs) are a significant concern at gas stations due to their impact on air quality and human health. It has been shown that petrol pump workers have the highest exposure to benzene, toluene, and xylenes.

Gas stations are one of the main sources of VOCs emission in urban air^11^. During refueling, VOCs are released as gasoline vapors escape into the air. Even with vapor recovery systems in place, some VOCs still manage to escape. VOCs can also be released from underground and above-ground storage tanks through venting and leaks. Additionally, accidental spills and leaks during fuel delivery and refueling contribute to VOC emissions. Vehicles idling or moving through the station also emit VOCs from their exhaust.

Many studies have been published on the emissions of volatile organic compounds (VOCs) at gas stations, but comparisons with natural gas stations or hybrid stations that offer both gasoline and natural gas have not been modeled.The objective of this study is to determine ambient concentrations of VOCs in three different types of refueling stations.

This study investigates gas stations from the point of view of environmental pollution. VOCs measurements were performed for three different kinds of gas stations located in Mashhad city to understand what type of gas station was responsible for the most pollution and what type of volatile organic compound emission is the most frequent.

## Materials and methods

### Study area

**Figure 1:**
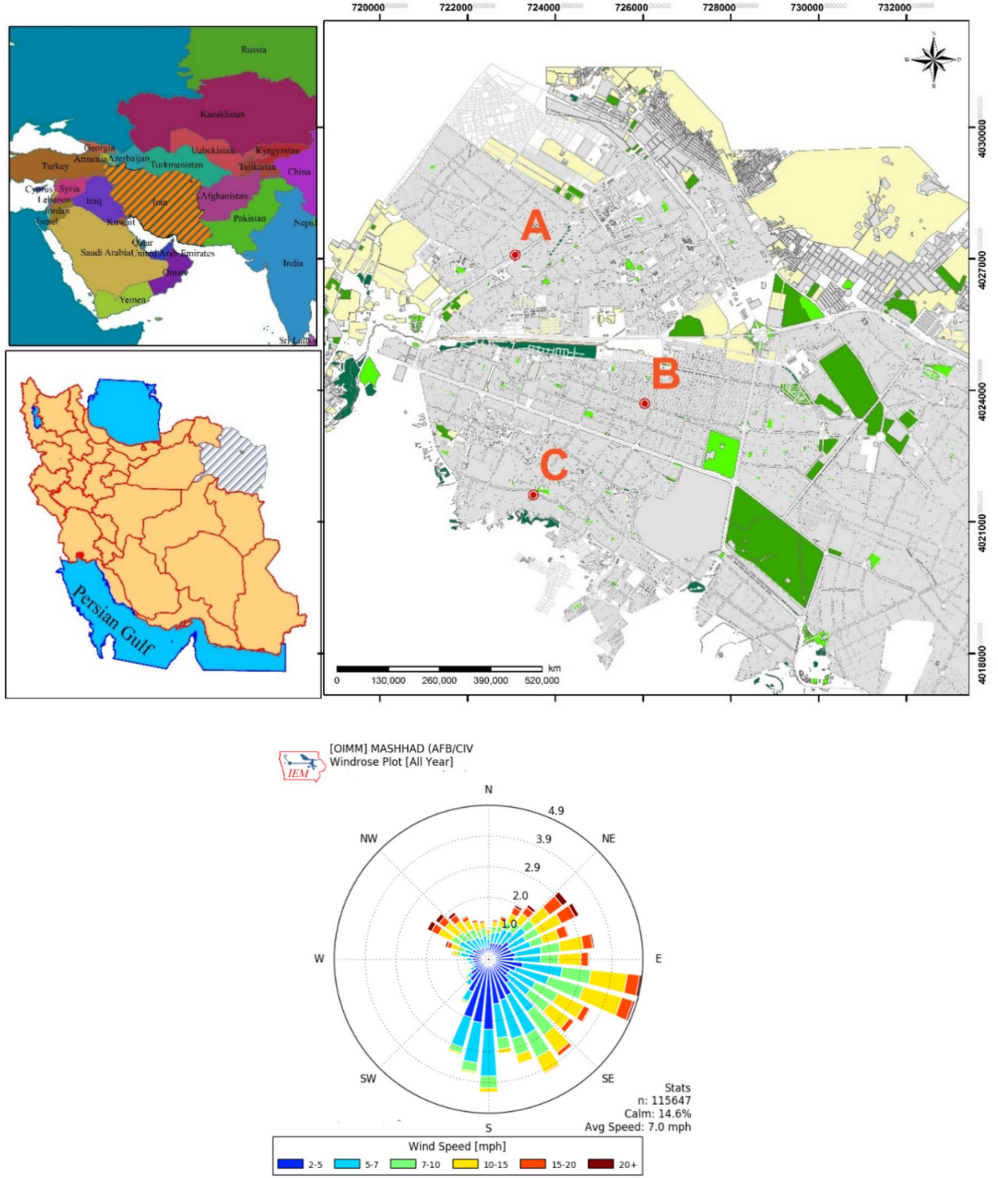
Location of sampling points in Mashhad city and windrose.

**Figure 2:**
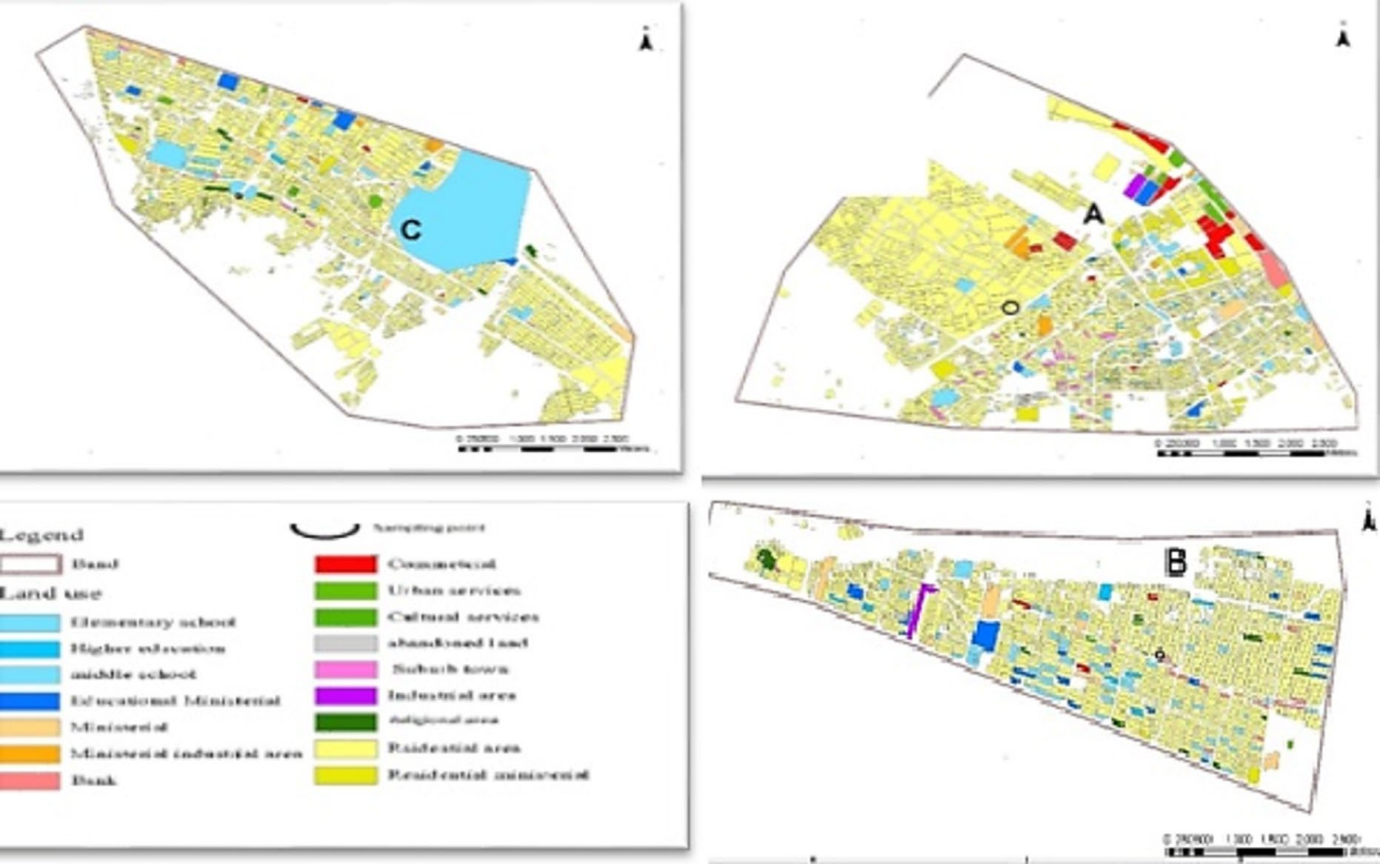
Description of the buildings and land use of sampling points in Mashhad.

Mashhad is the second most populous city in Iran with an area of 315 km^2^ and population of 3,372,660 people in 2016^12^. Moreover, Mashhad is the second largest religious city in the world such that more than 10 million pilgrims annually visit Mashhad. This city is one of the most polluted cities of Iran such that it has about 270–300 days of inversions per year due to the presence of numerous industrial plants and excessive heavy urban traffic in most parts of the city^13^. Air pollution in this city is responsible for some adverse health effects^14^. On average, 2.5 million liters of fuel are used daily by vehicles in Mashhad. A total of 20 filling stations owned by one company in the Mashhad were randomly selected for the study. These stations were selling CNG and gasoline. The sampling locations in the city of Mashhad and windrose are shown in Fig. 1. A detailed site map with the sample locations as well as a description of the buildings and land use in the vicinity are shown in Fig. 2. The purpose of this study was to investigate the effect of different types of fuel stations on the air quality of that area at the same time. The sampling was performed at three points in each station in the direction of wind (use of sonic anemometer and information about windrose): before (upstream of the fuel station) and after (downstream of the fueling station). The stations were not equipped with the system reduction, conduction, transportation, and gasoline vapor recovery. In Iran, the pressure/vacuum valves on vent pipes of fuel storage tanks are not common and used. Sampling from each station at every point was repeated 9 times. We repeated and kept on sampling until the standadd divition is little than one quartile.

There is a highway adjacent to Fuel Station A. This station commonly supplies both CNG and gasoline. Traffic flow is low at this location. Fuel Station B, which supplies gasoline fuel, is located in an area of the city subjected to heavy daily traffic. This site is close to many commercial and residential buildings and is also close to the main crossroads. Fuel Station C supplies CNG fuel and is located parallel to a main Boulevard in the city. The density of residential buildings is high in this area and the number of cars’ refuels in this station is relatively high. The stations are not equipped with system reduction, conduction, transportation, and gasoline vapor recovery. In Iran, the pressure/vacuum valves on vent pipes of fuel storage tanks are not common and are not used.

### Air sampling process and analyses

Meteorological data and other parameters related to traffic were also measured during the sampling (Table 2). The air temperature was measured using a thermometer and wind speed was measured with a sonic anemometer. The sampling and the analysis process for VOCs (BTEX) in ambient air were based on the EPA TO-17A method^15^. Sampling was carried out by the active sampling method using a hand-operated pump and charcoal sorbent tubes (SKC). Sampling was conducted for 2 h during peak filling times. The flow rate was regulated at 300 ml min^−1^ for 2 h and the measurement location was at a height of 1.5–2 m above ground level. Duration of sampling was based on the EPA TO-17A method and NIOSH Manual of Analytical. After sampling, the adsorption tubes were labeled and closed with special caps to avoid contamination and desorption. The samples were transferred to the laboratory immediately. The analysis was performed by a gas chromatograph (GC, Agilent 7890N, Agilent Co.) equipped with mass spectrometry (MS, Agilent 5975C, Agilent Co.). The analysis was performed according to the procedure presented by Sarkhosh et al.^16^.

## Results and discussion

### VOCs concentrations

**Figure 3:**
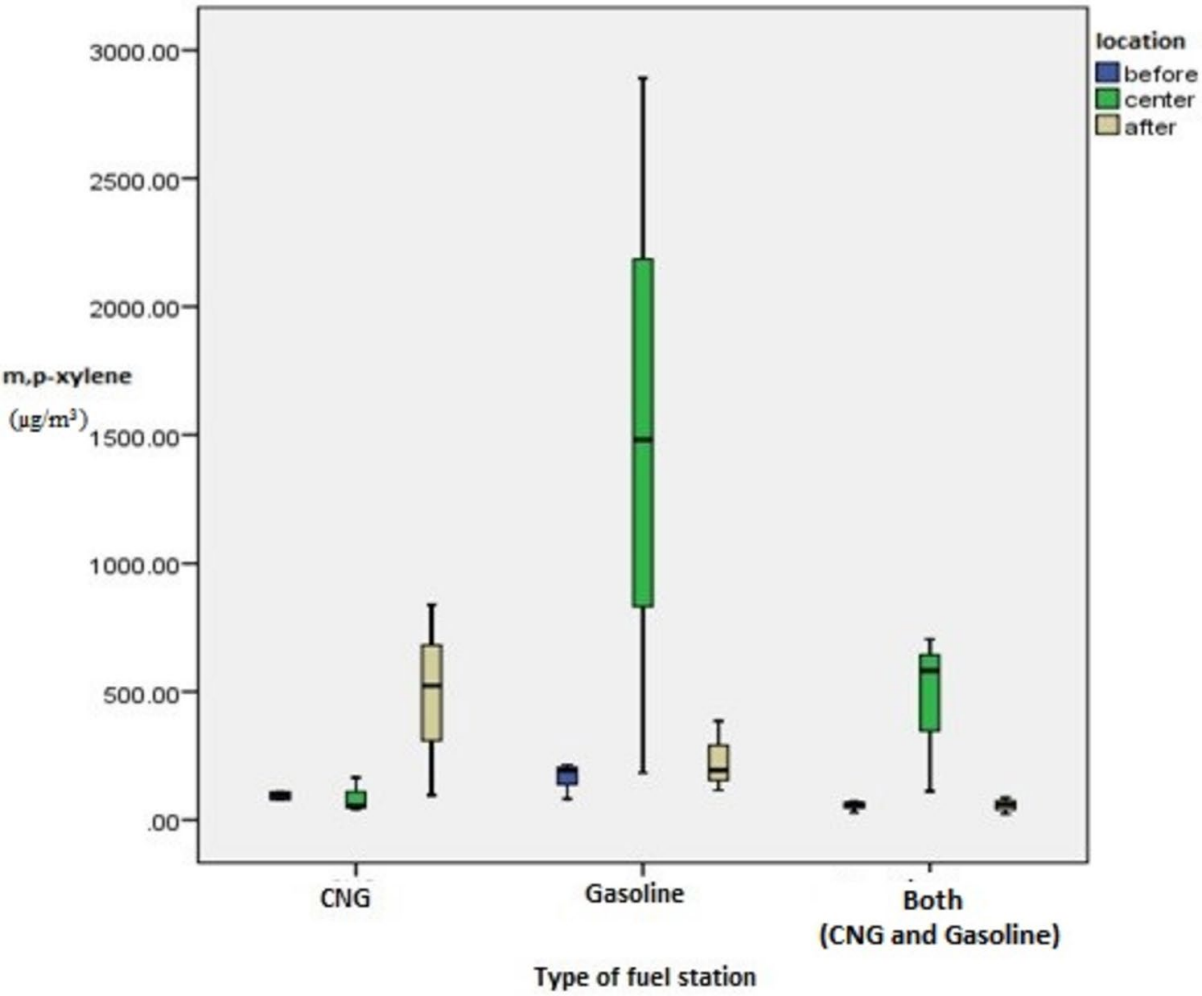
The box plot of m,p-xylene in different points at fuel stations.

The average VOCs concentration in each of the different gas stations is represented in Table 1. Total average VOCs measured during the sampling was 482.36 ± 563.45 µg m^−3^ at gas station A. At gas station B, the total average VOCs concentration was 1363.4 ± 1975 µg m^−3^ while it was 410.29 ± 483.37 µg m^−3^ at gas station C. The average total VOC concentration in site B was almost 3 times higher than that found in site A and site C. The majority of compounds identified were of the BTEX group. Xylene isomers and toluene were the most abundant compounds measured in all of the stations. The concentrations of m,p-xylene and toluene were respectively 46% and 18% at gas station B, 40% and 28% at gas station A, and 58% and 20% at gas station C. The lowest mean concentrations were observed for 1-ethyl-3-methyl benzene with amounts of 1.15 ± 0.34, 6.30 ± 12.64, and 1.27 ± 0.46 µg m^−3^ at gas stations A, B, and C, respectively. Figure 3 illustrates the amount of m,p-xylene in different points at the fuel stations.

**Table 1:** Average concentrations of VOCs at gas stations.

Location	code	VOCs concentration (µg m^−3^)								
		Benzene	Toluene	Ethylbenzene	m/p X	o-X	n-PB	1-eth -4	1-eth-3	1,2,3-tri
Gas station A	A_0_	1.00 ± 4.00	112.78 ± 165.61	23.15 ± 27.60	54.63 ± 21.73	80.00 ± 91.92	1.00 ± 4.00	1.00 ± 4.00	1.00 ± 4.00	1.00 ± 4.00
	A_1_	9.03 ± 12.67	241.76 ± 54.24	75.19 ± 36.41	466.39 ± 313.19	185.74 ± 129.14	9.76 ± 13.95	7.03 ± 10.98	0.00 ± 7.00	18.93 ± 16.33
	A_2_	1.00 ± 4.00	63.34 ± 76.53	12.13 ± 12.68	56.67 ± 32.35	16.11 ± 8.06	1.00 ± 4.00	1.00 ± 4.00	1.00 ± 4.00	1.00 ± 4.00
	Mean ± SD	3.70 ± 93.40	139.12 ± 29.18	36.37 ± 82.59	192.26 ± 56.99	93.11 ± 95.64	4.80 ± 17.14	3.60 ± 26.18	1.00 ± 15.34	7.11 ± 23.99
Gas station B	B_0_	1.00 ± 4.00	153.70 ± 182.53	29.26 ± 26.27	163.70 ± 71.34	58.89 ± 24.83	1.00 ± 4.00	1.00 ± 4.00	1.00 ± 4.00	1.00 ± 4.00
	B_1_	48.14 ± 82.19	486.67 ± 255.94	214.91 ± 147.08	1518.15 ± 1354.14	707.22 ± 697.90	78.83 ± 93.16	26.57 ± 44.82	16.15 ± 20.52	69.11 ± 76.29
	B_2_	9.99 ± 14.92	123.52 ± 102.07	53.15 ± 24.92	231.67 ± 138.90	88.15 ± 60.80	1.00 ± 4.00	1.00 ± 4.00	1.00 ± 4.00	1.00 ± 4.00
	Mean ± SD	19.47 ± 84.00	254.24 ± 63.35	99.12 ± 11.70	637.95 ± 84.36	284.47 ± 75.65	27.60 ± 20.58	9.25 ± 77.71	6.12 ± 30.64	23.51 ± 96.01
Gas station C	C_0_	6/48 ± 6.55	34.29 ± 43.43	6.22 ± 6.92	94.03 ± 21.01	38.06 ± 11.39	1.61 ± 0.33	1.61 ± 0.33	1.61 ± 0.33	1.61 ± 0.33
	C_1_	0.766 ± 0.132	84.53 ± 116.83	4.56 ± 2.89	87.41 ± 67.85	22.22 ± 5.63	0.76 ± 0.13	0.76 ± 0.13	0.76 ± 0.13	0.76 ± 0.13
	C_2_	1.53 ± 0.271	119.87 ± 116.60	55.00 ± 48.85	486.21 ± 372.91	99.07 ± 87.74	1.53 ± 0.27	1.53 ± 0.27	1.53 ± 0.27	1.53 ± 0.27
	Mean ± SD	2.48 ± 3.52	85.23 ± 96.49	23.89 ± 36.81	238.61 ± 288.37	55.00 ± 56.34	1.00 ± 27.46	1.00 ± 27.46	1.00 ± 27.46	1.00 ± 27.46

0 indicates before station, 1 indicates center of station and 2 indicates after of station.

*B* benzene, *T* toluene, *E* ethylbenzene, *m,P-x* m,p-xylene, *O-X* o-xylene, *n-PB* N-Propylbenzene, *1-eth-4* 1-ethyle-4-methyle benzene, *1-eth-3* 1-ethyle-3-methyle benzene, *1,2,3-tri* 1,2,3-trimethylebenzene.

The World Health Organization (WHO) established a threshold value of 260 µg m^−3^ as a weekly average concentration for toluene and 4800 µg m^−3^ as a 24-h average for xylenes^17^. The toluene content of gasoline and motor vehicle exhaust is higher than the benzene content^18^. M,p-xylene is found in petroleum derivatives such as petrol^19^. Also, toluene and xylene are added to gasoline to increase the octane rating^20^. It was confirmed that during the refueling process about 0.01% of fuel can be splashed and up to about 0.5% can be evaporated if equilibrated gasoline vapors during refueling are released from a tank to the ambient air^21^. Hilpert^22^ showed that emission pollutants from gas stations were at a very high rate (i.e., N10 times higher than estimates). According to Directive2000/69/EC, the annual mean benzene concentration in air may not exceed 5 µg m^−3^. The mean benzene values obtained in this study for gas stations A and C have always been below this limit. Higher benzene values were obtained for gas station B in some cases. The highest total VOCs concentration was recorded at station B.

The US EPA report was revealed that the concentrations of benzene (a carcinogen), toluene, ethylbenzene, and xylene as the general compounds of the gasoline are higher in 200 m within gas station than atmospheric background levels^23^. Yerushalmi & Rastan reported that in Canada, 11,200 retail gasoline outlets that entered 58 million liters of liquid gasoline into the atmosphere annually^23^. Due to the low background VOCs concentration (before station) and the increase in concentration after the gas station, it can be concluded that this station contributes to air pollution around the area. Karakitsios et al.^24^ indicated that gas stations are the main source of benzene emission. The concentrations of benzene diminished significantly as the distance from gas stations increased^24^. The type of unleaded fuels widely used in Asian cities contains a very high aromatic level (45%)^25^, while levels exceeding 35% are not allowed in EU member states^26^. Toluene and xylene values in the air of a large Italian city showed a decreasing trend. One reason for this result is the need for the reduction of aromatic hydrocarbon levels in Italian gasoline to 40%^27^. Suarez et al. reported that installing vapor recovery systems in Mexico City gas stations reduced significantly attendants’ exposure. The introduction of vapor recovery systems caused a reduction of 57% in xylene levels^28^. The values of BTEX monitored in the three gas stations in the city were comparable with those of fuel stations elsewhere^29–31^.

As noted above, m,p-xylene was the most abundant compound present in the three sites. The amount of this pollutant was higher than other compounds. However, in location C, where CNG fuel is used, the concentration of xylene was lower than at the other locations. In this regard, Naeem et al. showed that the declined VOCs, especially xylene emissions, in CNG fuel are related to the higher combustion temperature^32^. In other words, the lower content of aromatics and higher temperature in CNG vehicle engines leads to the decomposition of xylene and consequently, the amount of xylene in CNG vehicle exhaust decreases relative to gasoline.

Considering that xylene has a lifetime of only 7.8 h and that it does not remain in the atmosphere for a long time^33^, it can be concluded that xylene is produced constantly via evaporative emissions and vehicle exhausts, particularly from gasoline engines. Gasoline used in Iran contains higher levels of m,p-xylene^34^. One blend of petroleum contains 12.86 w/w% of this compound. An important application of this compound is its use as a solvent to remove organic materials in petroleum production^34^. Fuel combinations are among important factors that greatly affect the number of pollutant emissions from vehicles^35^. The existence of xylene in indoor and outdoor air can have deleterious effects on health human. The Massachusetts Department of Environmental Protection Agency has reported that the annual ambient limit for xylenes globally equals 12 µg m^−3^^7^.

### Modeling of m,p-xylene concentration

In this section, the time series analysis method is applied to the interpolation of m,p-xylene data with respect to time. This method has been applied in a study to model the daily nitric oxide (NO) concentrations measured at different stations^36^. The purpose of our modeling approach is to propose an equation that could capture the variation of the m,p-xylene over time.

In order to obtain high accuracy, we used interpolation with general equations. General (nonlinear) equations are defined as equations that are nonlinear in the parameters or are a combination of linear and nonlinear in the parameters. Interpolation is a method of finding new data points by the slope of the straight line between two know points. In this study, a combination of a sine function with a quadratic equation (a parabola) and a constant term is considered. A sine-function is defined since the periodic behavior of emissions has been reported in the literature^37^. The equation is defined as follows:

$$f\left( x \right) = a*\left( {sin\left( {x - \pi } \right)} \right) + b*\left( {\left( {x - 6} \right)^{2} } \right) + c$$

where parameters *a*, *b*, and *c* should be estimated such that the distance between *f*(*x*) and m,p-xylene to be minimized. The curve fitting method in MATALAB is applied for this purpose. Depending on the type of station—whether it is CNG, gasoline, or a combination—the coefficients A, B, and C will vary. The values of these coefficients can be found in Table 2.

**Table 2:** Parameters measured in the sampling points A, B, and C.

Parameter	Gas station A	Gas station B	Gas station C
Wind speed (m/s)	1.012 ± 0.36	1.82 ± 0.88	0.766 ± 0.36
Air temperature (°C)	32 ± 2.00	30 ± 1.15	28.33 ± 3.3
Relative humidity (%)	18.58 ± 0.95	21.33 ± 1.04	26.22 ± 0.96
Pressure (mmHg)	889.9 ± 0.34	893.25 ± 0.95	906.26 ± 36.85
Number of vehicle/min	34.33 ± 6.11	38 ± 3.61	26.33 ± 3.21
Number of refueling	127.33 ± 22.50	178 ± 21.38	183.67 ± 29.26
Vapor recovery technology	–	–	–
Number of nozzle spills	24	18	6
Dripless nozzles^1^	Yes	Yes	Yes
Fuel reserves (m^3^)	76	40	31
Ratio of CNG and gasoline	0.69	–	–

^1^Liquid trickle which happen during fueling by nozzle have been reduced by modified Dripless nozzles.

To determine the goodness of fit, the coefficients of the sum of squares due to error (SEE) and R square were calculated (Table 3).

**Table 3:** Logistic model coefficients for m,p-xylene.

		A	B	C	SSE	R-square
Fuel Station A	M,p-xylene	− 151.5	− 36.08	886.1	1.81e − 27	1
Fuel Station B	M,p-xylene	1015	− 36.7	1954	1.68e − 25	1
Fuel Station C	M,p-xylene	94.82	11.79	− 49.38	1.31e − 27	1

The results demonstrated that the combination of a sine function and a quadratic function could model the fluctuation behavior of air pollutants like m,p-xylene.

### Comparison of xylene and toluene levels measured with other studies

At gas stations, the concentrations of toluene and xylene are often the highest among the BTEX compounds. While these two compounds are not definitively carcinogenic, they can have harmful effects over the long term even at low concentrations. Table 4 shows concentrations of toluene and m,p-xylene emissions from fuel stations located in some cities of other countries. As you can see in the table below, other studies also show that xylene concentrations are higher than toluene concentrations. However, in Iran, this difference is much greater, which can be attributed to the quality and type of fuel. The differences in concentrations of these two compounds between the fuel stations can be related to the quality and type of fuel, vapor recovery technology, fuel reserves, dripless nozzles, traffic density in these stations, meteorological conditions and the location of sampling sites. Ambient concentrations of VOCs measured in the present study were comparable with those measured from refueling stations elsewhere. Studies conducted by Sehgal et al. showed that toluene and xylene concentrations were much higher during the dry season than the rainy season^38^.

**Table 4:** The average concentration of toluene and m,p-xylene in selected fuel stations around the world.

Studied by	Location	Toluene	M, p-xylene
This study	Mashhad (site 1)	139.29	192.56
	Mashhad (site 2)	254.63	637.84
	Mashhad (site 3)	85.23	238.61
Sehgal et al.^38^	Delhi	5890	9512
Esmaeel nejad et al.^39^	Shahreza	402.5	439
Rattanajongjitrakorn et al.^31^	Bangkok	1984	496
Correa et al.^29^	Rio de Janeiro	47.7	46.9

### Correlations and ratios between BTEX compounds

Spearman’s correlation of the concentrations of BTEX compounds was evaluated for the different sampling stations (Table 5).

**Table 5:** Spearman’s Correlation coefficients for the compounds studied.

Gas station A	Pollutant	Benzene	Toluene	Ethyl benzene	M, p-Xylene	O-xylene
	Benzene	1.000				
	Toluene	0.337	1.000			
	Ethyl benzene	0.495	0.95*	1.000		
	M, p-xylene	0.426	0.483	0.700*	1.000	
	O-xylene	0.495	0.8*	0.850*	0.65	1.000
Gas station B	Benzene	1.000				
	Toluene	− 0.18	1.000			
	Ethyl benzene	− 0.138	0.717*	1.000		
	M, p-xylene	0.203	0.410	0.669*	1.000	
	O-xylene	0.046	0.350	0.7*	0.929*	1.000
Gas station C	Benzene	1.000				
	Toluene	− 0.303	1.000			
	Ethyl benzene	0.303	− 0.119	1.000		
	M, p-xylene	0.376	0.238	0.748*	1.000	
	O-xylene	0.667	− 0.71	0.714*	0.833*	1.000

*Correlation is significant at the 0.05 level.

At gas stations A, B and C, a low correlation coefficient was found between benzene and the other TEX compounds measured. This is because the benzene content is the lowest (when compared with TEX) in the gasoline fuel, therefore, the evaporation of benzene to the atmosphere is the lowest^40^. xylene showed a good correlation with ethylbenzene. Ethylbenzene correlated well with toluene and o-xylene, suggesting that they most probably came from the same sources during the sampling (gas station).

Table 6 presents the m,p-X/EB, m,p-X/T, and m,p-X/B ratios for three studied stations. Ratios of the BTEX group was used as markers to determine emission sources.

**Table 6:** The ratio of BTEX species at three monitoring stations.

Monitoring stations	M,p-X/EB	M,p-X/T	M,p-X/B
A	5.22	1.38	48.99
B	12.01	2.50	32.13
C	9.98	2.79	96.21

The higher reaction rates for ethylbenzene and o-xylene with OH radicals result in their non-detection in urban and semi-rural areas^41^. Xylene is considered as the most reactive compound of the BTEX compounds^42^. The observed values of the m,p-X/EB ratio in this study are higher than those observed in other studies. The higher ratio of m,p-xylene/EB is related to the fuel stations^43^. A. Zalel et al. concluded that greater levels of m,p-xylene in the gasoline in Israel resulted in increased ratios involving m,p-xylene^44^. The higher values of m,p-X/B ratios indicate lower benzene concentration.

## Conclusions

The concentrations of ambient VOCs at three refueling stations with a different type of fuels in Mashhad were monitored. The study measured volatile organic compounds (VOCs) concentrations at three different gas stations (A, B, and C) and found significant variations among them. Gas station B exhibited the highest total average VOC concentration at 1363.4 ± 1975 µg m^−3^, which is nearly three times higher than the concentrations at stations A (482.36 ± 563.45 µg m^−3^) and C (410.29 ± 483.37 µg m^−3^). The BTEX group (benzene, toluene, ethylbenzene, and xylenes) was the most prevalent group of VOCs identified, with xylene isomers and toluene being the most abundant compounds at all stations. The result of this study showed that CNG fuel stations are less polluting than petrol stations.

Specifically, m,p-xylene and toluene constituted significant portions of the total VOC concentrations: 46% and 18% at station B, 40% and 28% at station A, and 58% and 20% at station C, respectively. The lowest mean concentrations were observed for 1-ethyl-3-methyl benzene, with values of 1.15 ± 0.34 µg m^−^3 at station A, 6.30 ± 12.64 µg m^−^3at station B, and 1.27 ± 0.46 µg.m-3 at station C. Overall, these results highlight the variability in VOC pollution across different fuel stations, with Gas Station B being a notable hotspot for higher VOC levels.

The study confirmed that VOC emissions during refueling can be substantial, contributing significantly to air pollution. The concentration of benzene, a known carcinogen, exceeded the European Directive 2000/69/EC annual mean limit of 5 µg m^−3^ at gas station B but remained below this limit at stations A and C. In all the studied sites, the highest concentrations were related to xylene isomers, irrespective of the fuel type. Presumably, this may be related to the constituents of the fuel used. Also, the quality of the fuel used plays a significant role in the concentration of pollutant emitted, especially that of xylene. These fuel stations are located close to residential buildings, thereby making the population vulnerable to exposure to these pollutants.

Comparative analysis showed that VOC concentrations near gas stations can be significantly higher than background atmospheric levels, as supported by studies from the US EPA and other research worldwide.

The presence of m,p-xylene was notably higher in the areas sampled, reflecting its higher concentration in Iranian gasoline. This study highlights the need for stricter controls and the implementation of vapor recovery technologies to mitigate the impact of VOCs on air quality, particularly in residential areas close to gas stations. Future research should continue to explore the effectiveness of different mitigation strategies and the long-term health impacts of exposure to VOCs from fuel stations. To deal with this situation, it is suggested applying mitigation measures in refueling stations, such as the installation of vapor recovery systems to diminish evaporative emissions, improvement of the fuel quality, and engine technology improvement in vehicles. Also, adopting the equipment can reduce fuel losses and reduce environmental cost and health risks.

## Data Availability

All data generated or analysed during this study are included in this published article.
